# Next-generation microbiology: from comparative genomics to gene function

**DOI:** 10.1186/s13059-021-02344-9

**Published:** 2021-04-29

**Authors:** Carolin M. Kobras, Andrew K. Fenton, Samuel K. Sheppard

**Affiliations:** 1 Department of Molecular Biology & Biotechnology, University of Sheffield, The Florey Institute for Host-Pathogen Interactions, Sheffield, UK; 2 Department of Biology & Biochemistry, University of Bath, Milner Centre for Evolution, Bath, UK

## Abstract

Microbiology is at a turning point in its 120-year history. Widespread next-generation sequencing has revealed genetic complexity among bacteria that could hardly have been imagined by pioneers such as Pasteur, Escherich and Koch. This data cascade brings enormous potential to improve our understanding of individual bacterial cells and the genetic basis of phenotype variation. However, this revolution in data science cannot replace established microbiology practices, presenting the challenge of how to integrate these new techniques. Contrasting comparative and functional genomic approaches, we evoke molecular microbiology theory and established practice to present a conceptual framework and practical roadmap for next-generation microbiology.

**Supplementary Information:**

The online version contains supplementary material available at 10.1186/s13059-021-02344-9.

## Introduction

Experimental approaches for studying bacteria have changed dramatically over the last 20 years [1]. Shifting in response to public interest and fuelled by technological advances, understanding of these remarkable organisms continues to rapidly advance. We now know more than ever before about the metabolism, environmental context and host interactions of microbes, and the rate of discovery shows little sign of slowing. Among the most influential shifts in technology has been the increasing use of large sequencing datasets in research practice. These contemporary research approaches continue to gain momentum, expanding into ever more ingenious ways of using sequencing data to discover complex patterns of behaviour and reach a deeper understanding of the bacterial cell. This rapid advancement has many conceptual benefits but has also come at a significant cost, as laboratories struggle to integrate these techniques and apply best research practices to new types of data.

The magnitude and complexity of large sequencing datasets can make them appear abstract to the non-specialist, potentially leading to subjective judgements about whether to believe the analyses or not. This can, in turn, risk general disenfranchisement of microbiology researchers away from genomic data, promoting an over-reliance on outside proofs or validations to give meaning to sequencing-based datasets. Here, we argue for an integrated future for microbiology that combines the strengths of traditional microbiology with the promise of emergent sequencing technologies. Addressing the widening gap in research practice, we discuss some of the most influential methodologies, the validation of findings from large sequencing datasets, and how comparative and functional genomics can be integrated to advance microbiology from fundamental discovery to contemporary microbiology research practice.

### Using a data deluge for qualitative and quantitative microbial genomics

It has been well over a decade since next-generation sequencing (NGS) platforms became widely available for microbial genomics. The cost of sequencing has continued to fall to a point where large sequence datasets are within the budget of most research groups. This democratisation of technology was not driven by a fundamental change in how DNA is sequenced. In fact, the major shift came through the upscaling of bridge amplification in the Illumina sequencing-by-synthesis process [2, 3]. This allowed the simultaneous sequencing of millions of individual DNA molecules in parallel by NGS machines generating huge amounts of data. While new single-molecule sequencing technologies developed by Oxford Nanopore and Pacific Biosystems gather momentum [4], the massively parallel Illumina NGS approach remains a major driver in the generation of large-scale DNA sequencing datasets. Key to the widespread use of NGS methodology are the diverse applications. Broadly, the functionality can be described under two contrasting modes. The first is a high-accuracy DNA sequencing function best applied on either de novo genome sequencing or making detailed comparisons between genomes. Here, the huge numbers of individual sequencing reads are combined to remove errors in base calling and generate high-confidence ensemble averages. The second mode is a counting function used to survey mixed populations of DNA or RNA molecules. Here, each sequencing read is examined individually, separated into groups and scored. This approach can, for example, be applied to measure the relative frequencies of mRNA levels in a cell or to capture the composition of a bacterial population from an environmental sample.

### Transformative sequencing technologies and the genetics of phenotype variation

Determining the genetic basis of phenotype variation is among the most pervasive aims in microbiology. This is a major challenge and requires understanding of how changes to genes, and their constituent DNA sequences, can alter gene function and affect a phenotypic change over time. Two of the most transformative techniques that address this in bacteria are genome-wide association studies (GWAS) [5–7] and transposon insertion sequencing methods (here referred to as Tn-Seq, but also known as HITS, InSeq or TraDIS) [8–11]. Both techniques are powered by NGS, but each uses different functions of DNA sequencing technologies. GWAS requires genomes from multiple strains within a population to identify genomic elements that are statistically associated with a given phenotype or environmental condition [12, 13] and therefore uses the high-accuracy function of NGS. In contrast, Tn-seq profiles fewer strains and uses the DNA counting function to identify transposon insertions in populations of mutants to identify the contribution each gene makes to bacterial survival within the specific experimental context [14, 15].

### Describing population-wide genomic variation

**Fig. 1:**
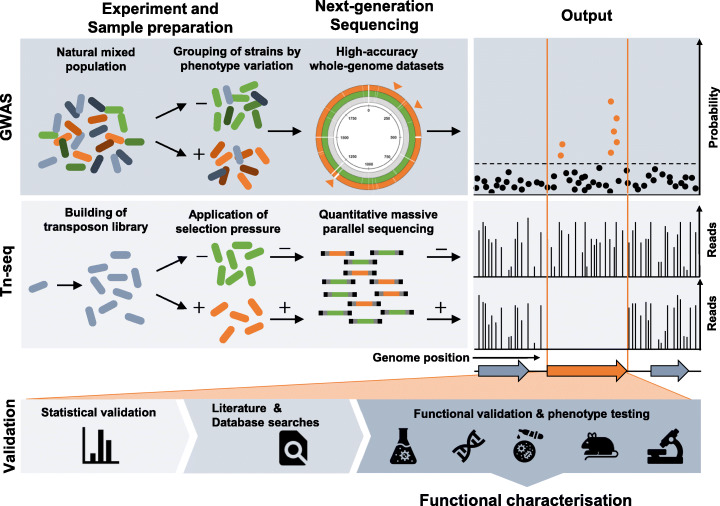
| Schematic overview of the GWAS and Tn-seq methods and a generalised validation pipeline. The gene highlighted in orange represents an idealised output for each approach. GWAS panels: In general, samples used for GWAS studies are directly isolated from the environment of interest. The phenotype of each isolate is tested and/or recorded, before whole-genome sequencing. Correlations between changes in observed genotypes and phenotype variations are determined. The output of GWAS can be displayed as a Manhattan plot, with the probability that each genetic variant detected in a population is associated with the phenotype of interest plotted against the genome positions. If variants fall above a certain probability threshold (dotted line), they are considered associated with the phenotype of interest (points highlighted in orange). Tn-seq panels: Saturated transposon libraries are grown in the presence and absence of the selection pressure of interest. Transposon-genome junctions from each member of the library are amplified and sequenced. Exploiting the quantitative function of massive parallel sequencing, the number of reads found for each transposon insertion junction are plotted against the genome position. The datasets obtained from libraries with and without the selection pressure are then compared to identify the contribution of each gene to the fitness. Areas of the genome with a different pattern of transposon insertions are deemed to be associated with the selection conditions (see region within the orange box). Validation panels: Initially the results of both methods are validated statistically and first insights into gene function are gained through literature and database searches. Deeper studies confirm the genotype-phenotype relationship of the results with a functional validation in the laboratory using a variety of experimental approaches

The availability of numerous high-quality bacterial genomes representing the extraordinary complexity of phenotypic and genotypic variation in natural populations has inevitably led microbiologists to new analytical techniques. Drawing on methods that were pioneered in human genetics, early bacterial GWAS approaches [5] have been adapted to become an important in silico tool for population-wide genomic screening [16]. Studies typically involve sampling and genome sequencing of hundreds of isolates from different environments or conditions and identifying genetic elements (e.g. single nucleotide polymorphisms (SNPs), k-mers or accessory genetic elements) that are significantly associated with a phenotype in question (Fig. 1). Now widely used, bacterial GWAS have successfully identified candidate genes involved in host specificity [5, 17], virulence [6, 18–24], the duration of pathogen carriage [25], and antibiotic resistance [7, 26–29].

The widespread application of bacterial GWAS has been made possible by adapting the methodological and analytical assumptions of human GWAS in two important ways. First, bacterial GWAS not only targets homologous sequence variation but also aims to identify the numerous accessory genetic elements and genes that may be found in some, but not all, isolate genomes [5, 30]. Second, and most importantly, it accounts for the strong linkage disequilibrium resulting from the clonal mode of bacterial reproduction. Accounting for this population structure is particularly important when considering the genetics underlying phenotype variation as causal variants will be co-inherited with linked loci that may have no adaptive function [12, 13]. In highly structured bacterial populations entire clusters of strains may share elements that have facilitated their expansion as well as those that simply reflect common ancestry. To address this, population subsampling [20, 31], linear mixed models [27, 32] and phylogenetic trees [21] can be incorporated into analyses to account for the clonal frame of the population. Resultant associations that cannot be explained by the effect of shared ancestry can represent convergent genomic signatures in groups of divergent strains. This provides clues to the evolutionary forces acting on the bacterial genome.

Sophisticated bioinformatics analyses of ever larger genome collections are: (i) incorporating quantitative trait variation [28]; (ii) conditioning on multiple genomic or phenotypic determinants [20, 29]; (iii) using machine learning to quantify the relative importance of associated elements in explaining the observed phenotype [20, 31, 33]. However, while bacterial GWAS approaches benefit from retaining the natural population setting of a given phenotype, they often return many thousands of genetic elements associated with complex traits such as host association or virulence [34]. In such cases, it can be extremely difficult to identify the role of individual genes and unravel the myriad interacting selective effects that shape the observed genomic variation. For this it may be necessary to move beyond in silico statistical associations and understand the function and importance of specific genes under more carefully controlled conditions.

### Studying gene function through modification and inactivation

Observing how small genomic differences, in otherwise isogenic strains, influence the phenotype provides evidence about the functional consequence of sequence variation. Over decades, microbiologists have identified the function of numerous genes across multiple species mainly through investigating the effect of gene loss. It is possible to infer gene function by inactivating specific genes, usually through introduction of a specific mutation into the genome of an organism and comparing the resultant phenotype to that of a ‘wild-type’ strain. While this is relatively laborious compared to observing genomic variation in natural populations in silico*,* it provides much greater control of the genomic variation and the conditions in which the gene function is being tested. Extending the principle of gene inactivation for genome-wide functional studies, ordered gene deletion libraries have been generated for several model laboratory strains [35–38]. In these libraries, all non-essential genes have been disrupted by the insertion of antibiotic markers, allowing the rapid screening of phenotypes under different selective conditions. Furthermore, random chemical or UV mutagenesis have been used to generate ordered mutant libraries, without requiring any a priori genetic manipulation of the bacterial strain [39]. While this allows investigations into bacterial species that are hard to manipulate genetically, it may be difficult to generate sufficient mutations for complete gene coverage in the screen, particularly as the whole genome of each strain needs to be sequenced to locate a mutation.

### Whole-genome fitness profiling using transposon insertion mutagenesis

Building on the concept of using large-scale gene deletion libraries that cover the entire genome of the bacterium, a revolution in these methodologies began just over a decade ago with the integration of quantitative high-throughput NGS technologies that capture the complexity of a large transposon-insertion library in one sequencing step [14, 15]. Developed around the same time, conceptually similar techniques including Tn-seq [8], TraDIS [9], HITS [10], and INSeq [11], all use large transposon insertion libraries, across which all or most non-essential genes contain transposon insertions. Selection pressure is applied to these library strains by growing them in defined in vitro or in vivo conditions (Fig. 1). Subsequent amplification and sequencing of the transposon-genome junctions in the libraries allows the insertion location of each transposon to be determined for each condition. The key feature of these approaches are the resulting ‘profiles’ of transposon insertions that reflect the fitness contribution each gene had under the selective conditions of the experiment. Specifically, regions of the genome where transposon insertions are statistically underrepresented likely contain genes that are essential for the bacteria to survive in the experimental conditions [8, 40–42].

This whole-genome fitness profiling method has linked many genes with metabolic pathways [43] and important phenotypes including stress response and antibiotic resistance [44–46], virulence and survival in the host environment [9–11, 47–51]. Furthermore, by deleting specific query genes it may be possible to identify gene interactions [8, 52, 53] and to examine the role of non-coding and regulatory DNA [54]. Most Tn-seq approaches rely on negative selection via gene inactivation. However, transposons carrying outward facing promoters can result in the upregulation of neighbouring genes. This allows controlled analysis of functional gene upregulation, an approach applied to the study of antibiotic resistance [55–57].

Recently, CRISPR interference (CRISPRi)-based methods have been added to the assortment of functional genomic tools [58]. Here, a small guide RNA forms a complex with the inactivated DNA-binding protein Cas9 and together they bind a specific region of the genome. The complex blocks RNA polymerase at the targeted site through steric hindrance, and represses transcription of the targeted gene [59, 60]. In a genome-wide screen, large libraries of mutants, each containing a different CRISPRi construct, can be captured by high-throughput NGS [61–66]. In contrast to Tn-seq, CRISPRi libraries have the potential of covering all genes in a genome, including essential genes. However, secondary and off-target effects still have to be carefully considered.

### Understanding bacteria in the wild

**Fig. 2:**
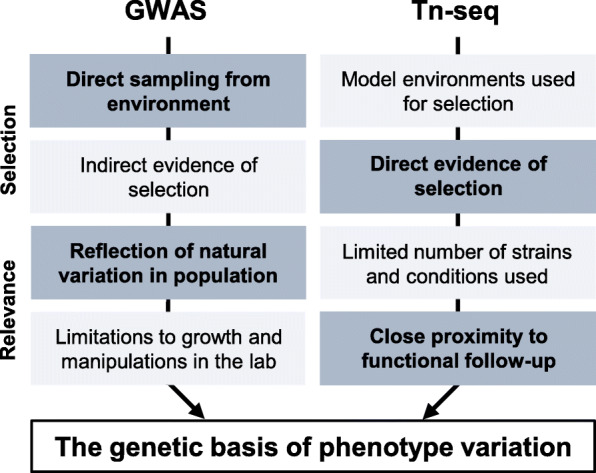
| GWAS and Tn-seq experimental approaches complement each other. Advantages (dark grey boxes) and limitations (light grey boxes) of both experimental approaches, focusing on differences in the application of selection pressure and relevance of the information gained from both methods

When trying to understand the genomics underlying trait variation in bacteria microbiologists must make compromises. The major challenge is to balance the experimental control needed to understand the function of specific genes with the requirement for data that is relevant in natural populations. This is illustrated by contrasting the selective conditions in Tn-Seq and GWAS approaches (Fig. 2). By deliberately limiting the selection pressures, Tn-seq studies provide a clear path to the functional validation of genes, often involving recreation of the initial experimental conditions used for the Tn-seq screen and measuring the fitness of genetically modified bacteria in competition-based assays [8, 44, 67]. However, in some cases there is surprisingly little overlap among the genes required for growth in particular conditions when comparing datasets between different laboratories [68]. This may be because of differences in: the precise experimental conditions; the transposons used; false-positives resulting from polar effects of transposon insertion; library selection and handling methods [69]. While the high-throughput nature of these methods, and appropriate validation, can largely overcome the challenge of reproducibility, the major strength of Tn-seq can also be considered its limitation. Specifically, while the deliberate constraint of the selection conditions acting on the bacteria facilitates functional genomics, these studies can also be criticised for lacking ‘real-world’ insight into genotype-phenotype relationships.

To enhance the relevance of laboratory findings for natural bacterial populations, recent multi-strain Tn-seq studies have included clinical or environmental isolates [42, 44, 46, 57]. However, there is a clear benefit to inference from bacteria in the wild. In this respect, GWAS is a powerful approach, as it directly surveys natural genotype-phenotype associations. This inevitably means that bacteria are sampled from dynamic systems and will have been exposed to a complex set of selection pressures, not all of which are directly related to the primary condition of interest. Structured sampling, replication and statistical tests, and in silico validations can strengthen assumptions about causal genetic variations [20], but recreating the experimental conditions in a laboratory setting may be extremely difficult, leading to difficulty when trying to understand the value of GWAS for lab-based microbiologists.

The complementary strengths and limitations of population-wide screens and laboratory fitness profiling methods provide a means to identify the genetic basis of complex bacterial traits (Fig. 2). Therefore, in combination, techniques such as GWAS and Tn-seq could provide insights into the behaviours of bacteria in natural environments in such a way as to be experimentally tractable in a laboratory setting. The initial steps taken to ensure data quality for both methods are similar, involving technical repeats and statistical validation. Yet, it is the experimental proof that specific genetic variants cause observable phenotypes that makes these studies so impactful. The challenges are how to achieve these proofs, what experimental methods should be used, and against what guidelines might we measure the evidence.

### Functional validation in the post-genomics era

**Fig. 3:**
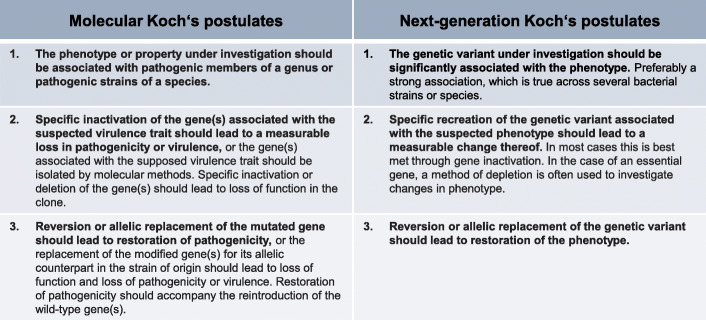
| Molecular Koch’s postulates, including a generalised revision for the purposes of this review. Stanley Falkow’s adaptation of Koch’s postulates (left) have been the gold standard to support causal links between genotypes and bacterial phenotypes for decades [71, 72]. To provide wider accessibility and application to microbiology research more generally, we have revised these postulates (right). Importantly, these postulates are adapted to guide functional validation and the authors acknowledge the full set might not be fulfilled in all cases

To the data analyst, functional genomic inference may be considered ‘validated’ if associations are proven robust against a series of statistical challenges. However, for laboratory-based researchers, in silico findings are typically considered ‘validated’ only when their effects can be reproduced using a complementary experimental approach. This requirement for experimental reproducibility has been a central tenet in microbiology since the publication of Koch’s postulates [70]. Adapting this conceptual framework in 1988, Stanley Falkow established a set of rules to prove causality of molecular genetic changes to disease phenotypes (Fig. 3) [70–72]. Subsequently adjusted to fit different research areas [73–76], these Molecular Koch’s postulates remain engrained in molecular microbiology best practice because of the scientific rigour they promote.

A major limitation of population-scale and genome-wide genetic screens is that a relatively small fraction of candidate genes are functionally validated. In some cases, in silico and laboratory-based genomic screens have employed follow-up gene inactivation and phenotype investigations to link the function of specific genes to pathogenicity [6, 18, 20, 31, 48, 77], survival and transmission [17, 19, 51] and antimicrobial resistance [29, 44, 57]. This experimental confirmation can be a challenging task given the large numbers of genes involved in complex phenotypes but it remains important for robust genotype-phenotype association. To address this we propose revised Molecular Koch’s postulates for functional genomic validation of NGS analyses (Fig. 3).

In most cases, genomic screening approaches will automatically fulfil the first postulate - that bacterial strains with an identified genetic variant should display the phenotype of interest (Fig. 3). Ideally, the relationship between genetic change and phenotype of interest would be as direct as possible, shifting only one experimental variable at a time and showing a large effect size. In this way, focusing on strong correlations can be useful as it provides the best experimental proofs.

According to the second postulate, specific changes made to the gene of interest should result in a change to the phenotype in question (Fig. 3). While not all genetic variants will result in the loss of the gene function, the generation of targeted gene deletions and reproduction of the experimental conditions leading to the expected phenotype is an approach often used to meet this postulate. To achieve this, tools for marked and markerless gene deletions have been developed [78–80], with the availability of ordered transposon or single-gene deletion libraries expediting this process for some bacterial species [35, 38, 81]. In cases where a gene is essential for the survival of a bacterium, depletion systems can be used but often require an established set of genetic tools to function. A popular example for sequence-specific repression of gene expression is CRISPRi [59, 60].

Finally, the third postulate focusses on restoring the observed phenotype through genetic complementation (Fig. 3). This is an essential step to close the loop and prove causation but may require a more sophisticated set of genetic tools to achieve. The most direct example is complementation via a conditional expression system, usually on a plasmid or at an ectopic locus in the genome. Alternatively, more subtle genetic manipulates can be used to meet this postulate, for example the introduction of a base pair change into the genome that complements the phenotype, an approach often achieved by site-directed mutagenesis [82].

The functional validation of genomic screens, potentially based on these revised Molecular Koch’s postulates, provides an ambitious target. In fact, it may be extremely difficult in practice to identify, remove and reinstate the genetics underlying trait variations. For example, where multiple independent variations cause subtle phenotypic changes or where genes are part of interactive networks and co-vary because of epistasis [83, 84]. As such, Molecular Koch’s postulates exist principally as a ‘gold standard’ rather than a definitive list of experimental criteria. In practice, integrated microbiology should layer multiple experimental approaches, using the minimum number of methods required to meet the burden of proof and focus validation effort for functional follow-up studies.

### A roadmap for next-generation functional microbiology

**Fig. 4:**
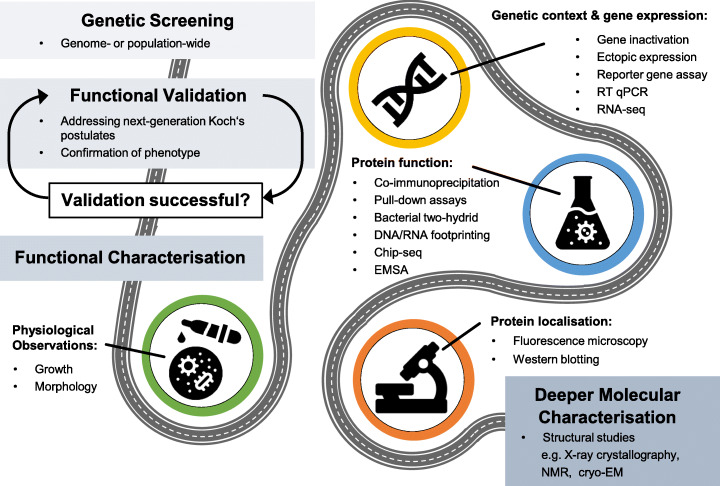
| A roadmap to understanding gene function. This figure splits the pathway of identifying, validating and investigating gene function into three different parts, with increasing depth of understanding. For the kinds of screening approaches discussed in this review, functional validation (top panel) is a crucial step in confirming the link between candidate genes and phenotype, which we argue should be carried out against criteria set out in next-generation Koch’s postulates. If successful, genes should be further functionally characterised towards deeper understanding (middle panel). This can be achieved using some of the methods laid out in the central panel and are shown here to guide researchers who are less familiar with these approaches. Deeper characterisations often require more specialist equipment and may be beyond the scope of non-specialist labs, we highlight a few examples here to place these types of methods in context (bottom panel)

Identifying and then proving that a genetic variant causes a phenotypic change is a considerable step towards understanding bacterial genomics. However, determining the precise role of the gene and how the encoded protein may function requires further study. This can be challenging, particularly for researchers with no background in molecular microbiology, even when the genetic methods or biochemical assays require fairly basic laboratory equipment. Therefore, just as laboratory microbiologists are encouraged to embrace contemporary genomic approaches, so bioinformaticians might consider the central role of functional microbiology in various ways (Fig. 4).

#### Physiological observations

Although considered rather simplistic, basic physiological observations are an important starting point for studies of gene function in microbiology. Differences in growth rate, signs of growth arrest, and early lysis are often tell-tale signs and should not be overlooked. Live-cell microscopy can also provide insights into whether a gene of interest affects cell morphology.

#### Genetic context and gene expression

In bacteria, genes with related functions often cluster together in operons. Therefore, deciphering the genetic context of candidate genes, as well as understanding where and when a gene is expressed in a bacterial cell, can provide insights into how they function. Determining the pattern and timing of gene expression can be accomplished by replacing the coding sequence of candidate genes with a reporter construct, allowing expression to be monitored through fluorescence, luminescence or enzymatic activity [85]. A more direct measure of gene expression is the determination of relative amounts of mRNA by reverse-transcription quantitative PCR (RT-PCR) [86]. Broadening this approach, techniques such as microarrays or RNA-seq represent powerful methods that may allow genome-wide functional transcriptomic analysis [87–89].

#### Protein function

When considering the biological role of a gene it is important to understand the function of the protein it encodes, especially if the protein is thought to have an enzymatic function or is likely to interact with binding partners in a wider network. This can include protein–protein interactions, protein–DNA/RNA binding, and other substrate or ligand interactions. Commonly used methods to identify protein-protein interactions are co-immunoprecipitation and protein pull-down assays. These conceptually similar approaches use antibodies to specifically recognise and isolate the (tagged) query protein from a cell lysate, bringing any binding partners with it [90]. Putative binding partners are then identified by mass spectrometry or other similar techniques. Alternative approaches include bacterial two-hybrid systems that are designed to report protein-protein interactions in vivo through expression of a reporter gene, most commonly beta-galactosidase or luciferase. This can be a very rapid approach to discovering or confirming individual predicted interactions, but may also be used to screen libraries of potential protein partners [91, 92].

Classically, DNA- or RNA-protein interactions are identified using DNA/RNA footprinting. Here, protein-bound DNA or RNA molecules are protected from cleavage by nuclease enzymes causing gaps in the digestion patterns when compared to DNA/RNA only controls [93]. More recently, genomic footprinting techniques based on NGS have been established, replacing the final gel separation steps with sequencing [94]. Genome-wide profiles of protein-DNA interactions can be further studied through combining chromatin immunoprecipitation with NGS (ChIP-seq) [95, 96]. Here, the query protein is fixed to the interacting DNA through chemical crosslinking in vivo. These complexes are enriched by immunoprecipitation and, after crosslink reversal, the DNA fragments are released and identified by NGS. ChIP-seq is especially useful for proteins with multiple binding sites, e.g. transcription factors. More focused methods for identifying specific protein-DNA/RNA binding, such as electrophoretic mobility shift assays (EMSA), exploit slower migration rates of protein-nucleic acid complexes in gels compared to nucleic acids alone [97]. Whatever the scale, protein interaction studies are essential for further molecular characterisation of genomic approaches, adding depth and context to candidate genes and advancing our understanding of the bacterial cell.

#### Protein localisation

Important clues to the specific function of a protein can be derived by examining its subcellular localisation. Separation of the bacterial cell into simple fractions (such as: membrane, cytoplasm, cell wall) can, in combination with western blotting, give a first indication. However, more specific insights into protein localisation can be achieved by fluorescence microscopy. This method usually involves the creation of protein-reporter fusions and allows tracking of the fluorescent product inside the cell [98–101]. In addition, protein tags, which are made fluorescent through the introduction of a small molecule, have become increasingly popular [102, 103]. While we highlight live-cell microscopy here, the expertise and special equipment required for microscopy methods beyond this quickly scale in complexity with the need for higher resolution.

#### Deeper molecular characterisation

The methods described so far should be practicable in most molecular microbiology laboratories. However, for many laboratories, this is where the research endeavour begins. For example, structural biologists acquire a deeper mechanistic understanding of protein function using methods such as X-ray crystallography [104], nuclear magnetic resonance (NMR) spectroscopy [105] and advanced optical methods such as (cryo-) electron microscopy [106], capitalising on the atomic resolution these methods bring.

### Examples for integrated next-generation microbiology

We have described a direction of travel for next-generation microbiology. Other researchers share the same vision and some studies have begun to bridge the gap and integrate large sequencing datasets with molecular microbiology. For example, early bacterial GWAS made simple comparisons between the putative function of the genes containing associated elements and basic bacterial growth assays with defined substrates [5]. In a more sophisticated approach, and consistent with next-generation Koch’s postulates, several studies have used mutagenesis and complementation to test the causality of hits and their predicted phenotype [6, 17, 18, 29, 51, 77]. Going still further, some studies have characterised the function of candidate genes and their genetic context [48, 107, 108], sometimes over multiple publications [84, 109]. For example, combining protein localisation microscopy with protein-protein interaction studies defined the role of candidate genes in the regulation of cell wall biosynthesis [52, 53]. Consistent with this, putative antimicrobial resistance determinants have been investigated in multiple bacterial strain backgrounds to provide information that is increasingly relevant to natural systems [44, 46, 57].

### Future directions

Microbiology research seeks to understand the workings of the bacterial cell in natural environments. Advances in DNA sequencing technologies have touched all aspects of microbiology research but this comes at a price. The specialism required to use these sequencing-based research methods risks a disconnect between bioinformatics and fundamental microbiology. This is because sequencing information is typically viewed through a series of analytical lenses to give it meaning. This means sequencing datasets are frequently abstract in nature and often the mathematical methods used to generate them are challenging to understand for non-specialists. With continued revisions to analytical methods and ever-increasing sample sizes, bioinformatic analysis has the potential to outpace fundamental microbiology investigations by orders of magnitude, exacerbating the analytical disconnect.

There are currently two principal solutions to this problem. First, bioinformaticians can collaborate widely, often contributing specific analytical expertise to each investigation. Second, bioinformatics in some areas has become a specialist data-service where researchers pay for analyses. In both cases, non-specialist researchers are divorced from the data, potentially leaning on internal controls or pre-assumed expectations to guide their interpretations. Similarly, bioinformaticians are removed from deeper understanding and characterisation of their initial discoveries. The typical conclusion is to argue for validated standardised analysis pipelines. This is sometimes necessary, such as in clinical settings, but in a rapidly evolving field it is vital that researchers are given flexibility for future innovation.

The pace of bioinformatics has moved microbiology research towards the study of natural populations. However, fundamental molecular microbiology approaches continue to focus on laboratory-adapted model strains for consistency across research groups. In the future, it will be important to merge these contrasting approaches to deepen the impact of research studies. Already, association studies (e.g. GWAS) or genome-wide profiles (e.g. Tn-seq) are commonly challenged to validate the gene function of at least one hit, while more fundamental laboratory studies are often compelled to contextualise their findings among more representative strains. In each case, this rising demand places strain on specialist laboratories. Here, we argue for an integrated future for next-generation microbiology, embracing new analysis techniques and placing in silico findings in a microbiological context. The spirit of integrated research is captured by large research collaborations or consortia but integration does not have to be collaboration in the strictest sense, it can be embodied by a small number of individuals who understand complementary research methodologies, provided they find a way to meet the burden of proof set out in next-generation Koch’s postulates. With careful reflection on current microbiology practice and a new awareness of the value of communicating complex datasets across disciplines, microbiology has never been in a better position to drive deeper understanding of the natural world.

## Supplementary Information

**Additional file 1.** Review history.

## References

[1] Bennett JW: Microbiology in the 21st Century. In: Designing the Microbial Research Commons: Proceedings of an Interantional Symposium. Volume 2. Edited by National Research Council (US) Board on Research Data and Information. Editor: P.F. Uhlir. Washington (DC): National Academies Press (US); 2011. 22593950

[2] Mardis ER (2008). Next-generation DNA sequencing methods. Annu Rev Genomics Hum Genet.

[3] Schuster SC (2008). Next-generation sequencing transforms today's biology. Nat Methods.

[4] Moss EL, Maghini DG, Bhatt AS (2020). Complete, closed bacterial genomes from microbiomes using nanopore sequencing. Nat Biotechnol.

[5] Sheppard SK, Didelot X, Meric G, Torralbo A, Jolley KA, Kelly DJ, et al. Genome-wide association study identifies vitamin B5 biosynthesis as a host specificity factor in *Campylobacter*. PNAS. 2013;110(29):11923–7. 10.1073/pnas.1305559110. 10.1073/pnas.1305559110 PMC3718156 23818615

[6] Laabei M, Recker M, Rudkin JK, Aldeljawi M, Gulay Z, Sloan TJ, et al. Predicting the virulence of MRSA from its genome sequence. Genome Res. 2014;24(5):839–49. 10.1101/gr.165415.113. 10.1101/gr.165415.113 PMC4009613 24717264

[7] Chewapreecha C, Marttinen P, Croucher NJ, Salter SJ, Harris SR, Mather AE, et al. Comprehensive identification of single nucleotide polymorphisms associated with beta-lactam resistance within pneumococcal mosaic genes. Plos Genet. 2014;10(8):e1004547. 10.1371/journal.pgen.1004547. 10.1371/journal.pgen.1004547 PMC4125147 25101644

[8] van Opijnen T, Bodi KL, Camilli A (2009). Tn-seq: high-throughput parallel sequencing for fitness and genetic interaction studies in microorganisms. Nat Methods.

[9] Langridge GC, Phan M-D, Turner DJ, Perkins TT, Parts L, Haase J, et al. Simultaneous assay of every *Salmonella typhi* gene using one million transposon mutants. Genome Res. 2009;19(12):2308–16. 10.1101/gr.097097.109. 10.1101/gr.097097.109 PMC2792183 19826075

[10] Gawronski JD, Wong SM, Giannoukos G, Ward DV, Akerley BJ (2009). Tracking insertion mutants within libraries by deep sequencing and a genome-wide screen for *Haemophilus* genes required in the lung. PNAS..

[11] Goodman AL, McNulty NP, Zhao Y, Leip D, Mitra RD, Lozupone CA (2009). Identifying genetic determinants needed to establish a human gut symbiont in its habitat. Cell Host Microbe.

[12] Read TD, Massey RC (2014). Characterizing the genetic basis of bacterial phenotypes using genome-wide association studies: a new direction for bacteriology. Genome Med.

[13] Chen PE, Shapiro BJ (2015). The advent of genome-wide association studies for bacteria. Curr Opin Microbiol.

[14] van Opijnen T, Camilli A (2013). Transposon insertion sequencing: a new tool for systems-level analysis of microorganisms. Nat Rev Microbiol..

[15] Cain AK, Barquist L, Goodman AL, Paulsen IT, Parkhill J, van Opijnen T (2020). A decade of advances in transposon-insertion sequencing. Nat Rev Genet..

[16] Power RA, Parkhill J, de Oliveira T (2017). Microbial genome-wide association studies: lessons from human GWAS. Nat Rev Genet.

[17] Yahara K, Méric G, Taylor AJ, de Vries SP, Murray S, Pascoe B (2017). Genome-wide association of functional traits linked with *Campylobacter jejuni* survival from farm to fork. Environ Microbiol.

[18] Laabei M, Uhlemann A-C, Lowy FD, Austin ED, Yokoyama M, Ouadi K, et al. Evolutionary trade-offs underlie the multi-faceted virulence of *Staphylococcus aureus*. Plos Biol. 2015;13(9):e1002229. 10.1371/journal.pbio.1002229. 10.1371/journal.pbio.1002229 PMC4558032 26331877

[19] Pascoe B, Méric G, Murray S, Yahara K, Mageiros L, Bowen R, et al. Enhanced biofilm formation and multi-host transmission evolve from divergent genetic backgrounds in *Campylobacter jejuni*. Environ Microbiol. 2015;17(11):4779–89. 10.1111/1462-2920.13051. 10.1111/1462-2920.13051 PMC4862030 26373338

[20] Méric G, Mageiros L, Pensar J, Laabei M, Yahara K, Pascoe B, et al. Disease-associated genotypes of the commensal skin bacterium *Staphylococcus epidermidis*. Nat Commun. 2018;9(1):5034. 10.1038/s41467-018-07368-7. 10.1038/s41467-018-07368-7 PMC6261936 30487573

[21] Collins C, Didelot X (2018). A phylogenetic method to perform genome-wide association studies in microbes that accounts for population structure and recombination. Plos Comp Biol.

[22] Berthenet E, Yahara K, Thorell K, Pascoe B, Meric G, Mikhail JM, et al. A GWAS on *Helicobacter pylori* strains points to genetic variants associated with gastric cancer risk. BMC Biol. 2018;16(1):84. 10.1186/s12915-018-0550-3. 10.1186/s12915-018-0550-3 PMC6090961 30071832

[23] Lees JA, Ferwerda B, Kremer PHC, Wheeler NE, Serón MV, Croucher NJ, et al. Joint sequencing of human and pathogen genomes reveals the genetics of pneumococcal meningitis. Nat Commun. 2019;10(1):2176. 10.1038/s41467-019-09976-3. 10.1038/s41467-019-09976-3 PMC6520353 31092817

[24] Kachroo P, Eraso JM, Beres SB, Olsen RJ, Zhu L, Nasser W, et al. Integrated analysis of population genomics, transcriptomics and virulence provides novel insights into *Streptococcus pyogenes* pathogenesis. Nat Genet. 2019;51(3):548–59. 10.1038/s41588-018-0343-1. 10.1038/s41588-018-0343-1 PMC8547240 30778225

[25] Lees JA, Croucher NJ, Goldblatt D, Nosten F, Parkhill J, Turner C, et al. Genome-wide identification of lineage and locus specific variation associated with pneumococcal carriage duration. eLife. 2017;6:e26255. 10.7554/eLife.26255. 10.7554/eLife.26255 PMC5576492 28742023

[26] Farhat MR, Shapiro BJ, Kieser KJ, Sultana R, Jacobson KR, Victor TC, et al. Genomic analysis identifies targets of convergent positive selection in drug-resistant *Mycobacterium tuberculosis*. Nat Genet. 2013;45(10):1183–9. 10.1038/ng.2747. 10.1038/ng.2747 PMC3887553 23995135

[27] Earle SG, Wu CH, Charlesworth J, Stoesser N, Gordon NC, Walker TM, et al. Identifying lineage effects when controlling for population structure improves power in bacterial association studies. Nat Microbiol. 2016;1(5):16041. 10.1038/nmicrobiol.2016.41. 10.1038/nmicrobiol.2016.41 PMC5049680 27572646

[28] Farhat MR, Freschi L, Calderon R, Ioerger T, Snyder M, Meehan CJ, et al. GWAS for quantitative resistance phenotypes in *Mycobacterium tuberculosis* reveals resistance genes and regulatory regions. Nat Commun. 2019;10(1):2128. 10.1038/s41467-019-10110-6. 10.1038/s41467-019-10110-6 PMC6513847 31086182

[29] Ma KC, Mortimer TD, Duckett MA, Hicks AL, Wheeler NE, Sánchez-Busó L, et al. Increased power from conditional bacterial genome-wide association identifies macrolide resistance mutations in *Neisseria gonorrhoeae*. Nat Commun. 2020;11(1):5374. 10.1038/s41467-020-19250-6. 10.1038/s41467-020-19250-6 PMC7584619 33097713

[30] Lees JA, Galardini M, Bentley SD, Weiser JN (2018). Corander J: pyseer: a comprehensive tool for microbial pangenome-wide association studies. Bioinformatics..

[31] Mageiros LMG, Bayliss SC, Pensar J, Pascoe B, Mourkas E, Calland JK, et al. Genome evolution and emergence of pathogenicity in avian *Escherichia coli*. Nat Commun. 2021;12:1–13 10.1038/s41467-021-20988-w PMC7858641 33536414

[32] Lees JA, Vehkala M, Välimäki N, Harris SR, Chewapreecha C, Croucher NJ, et al. Sequence element enrichment analysis to determine the genetic basis of bacterial phenotypes. Nat Commun. 2016;7(1):12797. 10.1038/ncomms12797. 10.1038/ncomms12797 PMC5028413 27633831

[33] Lees JA, Mai TT, Galardini M, Wheeler NE, Horsfield ST, Parkhill J (2020). Improved prediction of bacterial genotype-phenotype associations using interpretable pangenome-spanning regressions. mBio.

[34] Sheppard SK, Guttman DS, Fitzgerald JR (2018). Population genomics of bacterial host adaptation. Nat Rev Genet..

[35] Baba T, Ara T, Hasegawa M, Takai Y, Okumura Y, Baba M (2006). Construction of *Escherichia coli* K-12 in-frame, single-gene knockout mutants: the Keio collection. Mol Syst Biol.

[36] de Berardinis V, Vallenet D, Castelli V, Besnard M, Pinet A, Cruaud C, et al. A complete collection of single-gene deletion mutants of *Acinetobacter baylyi* ADP1. Mol Syst Biol. 2008;4(1):174. 10.1038/msb.2008.10. 10.1038/msb.2008.10 PMC2290942 18319726

[37] Porwollik S, Santiviago CA, Cheng P, Long F, Desai P, Fredlund J, et al. Defined single-gene and multi-gene deletion mutant collections in *Salmonella enterica* vs *typhimurium*. Plos One. 2014;9(7):e99820. 10.1371/journal.pone.0099820. 10.1371/journal.pone.0099820 PMC4089911 25007190

[38] Koo BM, Kritikos G, Farelli JD, Todor H, Tong K, Kimsey H, et al. Construction and analysis of two genome-scale deletion libraries for *Bacillus subtilis*. Cell Syst. 2017;4(3):291–305 e297. 10.1016/j.cels.2016.12.013. 10.1016/j.cels.2016.12.013 PMC5400513 28189581

[39] Kokes M, Dunn JD, Granek JA, Nguyen BD, Barker JR, Valdivia RH, et al. Integrating chemical mutagenesis and whole-genome sequencing as a platform for forward and reverse genetic analysis of *Chlamydia*. Cell Host Microbe. 2015;17(5):716–25. 10.1016/j.chom.2015.03.014. 10.1016/j.chom.2015.03.014 PMC4418230 25920978

[40] Remmele CW, Xian Y, Albrecht M, Faulstich M, Fraunholz M, Heinrichs E, et al. Transcriptional landscape and essential genes of *Neisseria gonorrhoeae*. Nucleic Acids Res. 2014;42(16):10579–95. 10.1093/nar/gku762. 10.1093/nar/gku762 PMC4176332 25143534

[41] Goodall ECA, Robinson A, Johnston IG, Jabbari S, Turner KA, Cunningham AF, et al. The essential genome of *Escherichia coli* K-12. mBio. 2018;9:e02096–17. 10.1128/mBio.02096-17 PMC5821084 29463657

[42] Poulsen BE, Yang R, Clatworthy AE, White T, Osmulski SJ, Li L, et al. Defining the core essential genome of *Pseudomonas aeruginosa*. PNAS. 2019;116(20):10072–80. 10.1073/pnas.1900570116. 10.1073/pnas.1900570116 PMC6525520 31036669

[43] Wetmore KM, Price MN, Waters RJ, Lamson JS, He J, Hoover CA (2015). Rapid quantification of mutant fitness in diverse bacteria by sequencing randomly bar-coded transposons. mBio.

[44] van Opijnen T, Dedrick S, Bento J (2016). Strain dependent genetic networks for antibiotic-sensitivity in a bacterial pathogen with a large pan-genome. Plos Path.

[45] Price MN, Wetmore KM, Waters RJ, Callaghan M, Ray J, Liu H, et al. Mutant phenotypes for thousands of bacterial genes of unknown function. Nature. 2018;557(7706):503–9. 10.1038/s41586-018-0124-0. 10.1038/s41586-018-0124-0 29769716

[46] Carey AF, Rock JM, Krieger IV, Chase MR, Fernandez-Suarez M, Gagneux S, et al. TnSeq of *Mycobacterium tuberculosis* clinical isolates reveals strain-specific antibiotic liabilities. Plos Path. 2018;14(3):e1006939. 10.1371/journal.ppat.1006939. 10.1371/journal.ppat.1006939 PMC5854444 29505613

[47] van Opijnen T, Camilli A (2012). A fine scale phenotype-genotype virulence map of a bacterial pathogen. Genome Res.

[48] Fu Y, Waldor MK, Mekalanos JJ (2013). Tn-Seq analysis of *Vibrio cholerae* intestinal colonization reveals a role for T6SS-mediated antibacterial activity in the host. Cell Host Microbe.

[49] Turner KH, Wessel AK, Palmer GC, Murray JL, Whiteley M (2015). Essential genome of *Pseudomonas aeruginosa* in cystic fibrosis sputum. PNAS..

[50] Duncan MC, Gillette RK, Maglasang MA, Corn EA, Tai AK, Lazinski DW, et al. High-throughput analysis of gene function in the bacterial predator *Bdellovibrio bacteriovorus*. mBio. 2019;10:e01040–19. 10.1128/mBio.01040-19 PMC6561027 31186325

[51] Rowe HM, Karlsson E, Echlin H, Chang T-C, Wang L, van Opijnen T (2019). Bacterial factors required for transmission of *Streptococcus pneumoniae* in mammalian hosts. Cell Host Microbe.

[52] Fenton AK, El Mortaji L, Lau DTC, Rudner DZ, Bernhardt TG (2016). CozE is a member of the MreCD complex that directs cell elongation in *Streptococcus pneumoniae*. Nat Microbiol.

[53] Fenton AK, Manuse S, Flores-Kim J, Garcia PS, Mercy C, Grangeasse C, et al. Phosphorylation-dependent activation of the cell wall synthase PBP2a in *Streptococcus pneumoniae* by MacP. PNAS. 2018;115(11):2812–7. 10.1073/pnas.1715218115. 10.1073/pnas.1715218115 PMC5856526 29487215

[54] Christen B, Abeliuk E, Collier JM, Kalogeraki VS, Passarelli B, Coller JA, et al. The essential genome of a bacterium. Mol Syst Biol. 2011;7(1):528. 10.1038/msb.2011.58. 10.1038/msb.2011.58 PMC3202797 21878915

[55] Wang H, Claveau D, Vaillancourt JP, Roemer T, Meredith TC (2011). High-frequency transposition for determining antibacterial mode of action. Nat Chem Biol.

[56] Santiago M, Lee W, Fayad AA, Coe KA, Rajagopal M, Do T, et al. Genome-wide mutant profiling predicts the mechanism of a lipid II binding antibiotic. Nat Chem Biol. 2018;14(6):601–8. 10.1038/s41589-018-0041-4. 10.1038/s41589-018-0041-4 PMC5964011 29662210

[57] Coe KA, Lee W, Stone MC, Komazin-Meredith G, Meredith TC, Grad YH, et al. Multi-strain Tn-Seq reveals common daptomycin resistance determinants in *Staphylococcus aureus*. PLoS Pathog. 2019;15(11):e1007862. 10.1371/journal.ppat.1007862. 10.1371/journal.ppat.1007862 PMC6934316 31738809

[58] Todor H, Silvis MR, Osadnik H, Gross CA (2021). Bacterial CRISPR screens for gene function. Curr Opin Microbiol.

[59] Wiedenheft B, Sternberg SH, Doudna JA (2012). RNA-guided genetic silencing systems in bacteria and archaea. Nature..

[60] Larson MH, Gilbert LA, Wang X, Lim WA, Weissman JS, Qi LS (2013). CRISPR interference (CRISPRi) for sequence-specific control of gene expression. Nat Protoc.

[61] Peters JM, Colavin A, Shi H, Czarny TL, Larson MH, Wong S, et al. A comprehensive, CRISPR-based functional analysis of essential genes in bacteria. Cell. 2016;165(6):1493–506. 10.1016/j.cell.2016.05.003. 10.1016/j.cell.2016.05.003 PMC4894308 27238023

[62] Wang T, Guan C, Guo J, Liu B, Wu Y, Xie Z, et al. Pooled CRISPR interference screening enables genome-scale functional genomics study in bacteria with superior performance. Nat Commun. 2018;9(1):2475. 10.1038/s41467-018-04899-x. 10.1038/s41467-018-04899-x PMC6018678 29946130

[63] Lee HH, Ostrov N, Wong BG, Gold MA, Khalil AS, Church GM (2019). Functional genomics of the rapidly replicating bacterium *Vibrio natriegens* by CRISPRi. Nat Microbiol.

[64] Liu X, Kimmey JM, Matarazzo L, de Bakker V, Van Maele L, Sirard J-C, et al. Exploration of bacterial bottlenecks and *Streptococcus pneumoniae* pathogenesis by CRISPRi-Seq. Cell Host Microbe. 2020;29:107–120.e106. 10.1016/j.chom.2020.10.001 PMC7855995 33120116

[65] Hawkins JS, Silvis MR, Koo B-M, Peters JM, Osadnik H, Jost M (2020). Mismatch-CRISPRi reveals the co-varying expression-fitness relationships of essential genes in *Escherichia coli* and *Bacillus subtilis*. Cell Syst.

[66] Jiang W, Oikonomou P, Tavazoie S (2020). Comprehensive genome-wide perturbations via CRISPR adaptation reveal complex genetics of antibiotic sensitivity. Cell.

[67] Kamp HD, Patimalla-Dipali B, Lazinski DW, Wallace-Gadsden F, Camilli A (2013). Gene fitness landscapes of *Vibrio cholerae* at important stages of its life cycle. Plos Path..

[68] Lee SA, Gallagher LA, Thongdee M, Staudinger BJ, Lippman S, Singh PK, et al. General and condition-specific essential functions of *Pseudomonas aeruginosa*. PNAS. 2015;112(16):5189–94. 10.1073/pnas.1422186112. 10.1073/pnas.1422186112 PMC4413342 25848053

[69] Burby PE, Nye TM, Schroeder JW, Simmons LA (2017). Implementation and data analysis of Tn-seq, whole-genome resequencing, and single-molecule real-time sequencing for bacterial genetics. J Bacteriol.

[70] Koch R: Über bakteriologische Forschung. In: Verhandlungen des X. Internationalen Medizinischen Kongresses, Berlin 1890. Volume 1. Berlin: Verlag von August Hirschwald; 1892.

[71] Falkow S (1988). Molecular Koch's postulates applied to microbial pathogenicity. Rev Infect Dis.

[72] Falkow S (2004). Molecular Koch's postulates applied to bacterial pathogenicity--a personal recollection 15 years later. Nat Rev Microbiol..

[73] Evans AS (1976). Causation and disease: the Henle-Koch postulates revisited. Yale J Biol Med.

[74] Fredericks DN, Relman DA (1996). Sequence-based identification of microbial pathogens: a reconsideration of Koch's postulates. Clin Microbiol Rev.

[75] Gradmann C (2014). A spirit of scientific rigour: Koch's postulates in twentieth-century medicine. Microb Infect.

[76] Byrd AL, Segre JA (2016). Adapting Koch's postulates. Science..

[77] Mourkas E, Taylor AJ, Méric G, Bayliss SC, Pascoe B, Mageiros L, et al. Agricultural intensification and the evolution of host specialism in the enteric pathogen *Campylobacter jejuni*. PNAS. 2020;117(20):11018–28. 10.1073/pnas.1917168117. 10.1073/pnas.1917168117 PMC7245135 32366649

[78] Thomason LC, Sawitzke JA, Li X, Costantino N, Court DL (2014). Recombineering: genetic engineering in Bacteria using homologous recombination. Curr Protocols Mol Biol.

[79] Selle K, Barrangou R (2015). Harnessing CRISPR–Cas systems for bacterial genome editing. Trends Microbiol.

[80] McClure EE, Chávez ASO, Shaw DK, Carlyon JA, Ganta RR, Noh SM (2017). Engineering of obligate intracellular bacteria: progress, challenges and paradigms. Nat Rev Microbiol.

[81] Fey PD, Endres JL, Yajjala VK, Widhelm TJ, Boissy RJ, Bose JL (2013). A genetic resource for rapid and comprehensive phenotype screening of nonessential *Staphylococcus aureus* genes. mBio.

[82] Ho SN, Hunt HD, Horton RM, Pullen JK, Pease LR (1989). Site-directed mutagenesis by overlap extension using the polymerase chain reaction. Gene..

[83] Arnold BJ, Gutmann MU, Grad YH, Sheppard SK, Corander J, Lipsitch M, et al. Weak epistasis may drive adaptation in recombining bacteria. Genetics. 2018;208(3):1247–60. 10.1534/genetics.117.300662. 10.1534/genetics.117.300662 PMC5844334 29330348

[84] Yokoyama M, Stevens E, Laabei M, Bacon L, Heesom K, Bayliss S, et al. Epistasis analysis uncovers hidden antibiotic resistance-associated fitness costs hampering the evolution of MRSA. Genome Biol. 2018;19(1):94. 10.1186/s13059-018-1469-2. 10.1186/s13059-018-1469-2 PMC6052701 30021593

[85] Bronstein I, Fortin J, Stanley PE, Stewart GS, Kricka LJ (1994). Chemiluminescent and bioluminescent reporter gene assays. Anal Biochem.

[86] Nolan T, Hands RE, Bustin SA (2006). Quantification of mRNA using real-time RT-PCR. Nat Protoc.

[87] Croucher NJ, Thomson NR (2010). Studying bacterial transcriptomes using RNA-seq. Curr Opin Microbiol.

[88] Sorek R, Cossart P (2010). Prokaryotic transcriptomics: a new view on regulation, physiology and pathogenicity. Nat Rev Genet..

[89] Saliba A-E, C Santos S, Vogel J: New RNA-seq approaches for the study of bacterial pathogens. Curr Opin Microbiol 2017;35**:**78–87, doi: 10.1016/j.mib.2017.01.001. 10.1016/j.mib.2017.01.001 28214646

[90] Lin J-S, Lai E-M, Journet L, Cascales E (2017). Protein–Protein Interactions: Co-Immunoprecipitation. Bacterial Protein Secretion Systems: Methods and Protocols.

[91] Karimova G, Pidoux J, Ullmann A, Ladant D (1998). A bacterial two-hybrid system based on a reconstituted signal transduction pathway. PNAS..

[92] Karimova G, Gauliard E, Davi M, Ouellette SP, Ladant D, Journet L, Cascales E (2017). Protein–Protein Interaction: Bacterial Two-Hybrid. Bacterial Protein Secretion Systems: Methods and Protocols.

[93] Galas DJ, Schmitz A (1978). DNAse footprinting: a simple method for the detection of protein-DNA binding specificity. Nucleic Acids Res.

[94] Vierstra J, Stamatoyannopoulos JA (2016). Genomic footprinting. Nat Methods.

[95] Wade JT, Struhl K, Busby SJW, Grainger DC (2007). Genomic analysis of protein–DNA interactions in bacteria: insights into transcription and chromosome organization. Mol Microbiol.

[96] Bonocora RP, Wade JT, Artsimovitch I, Santangelo TJ (2015). ChIP-Seq For Genome-Scale Analysis Of Bacterial DNA-Binding Proteins. Bacterial Transcriptional Control: Methods and Protocols.

[97] Hellman LM, Fried MG (2007). Electrophoretic mobility shift assay (EMSA) for detecting protein-nucleic acid interactions. Nat Protoc.

[98] Phillips GJ (2001). Green fluorescent protein – a bright idea for the study of bacterial protein localization. FEMS Microbiol Lett.

[99] Lippincott-Schwartz J, Patterson GH (2003). Development and use of fluorescent protein markers in living cells. Science..

[100] Shaner NC, Steinbach PA, Tsien RY (2005). A guide to choosing fluorescent proteins. Nat Methods.

[101] Wang S, Moffitt JR, Dempsey GT, Xie XS, Zhuang X (2014). Characterization and development of photoactivatable fluorescent proteins for single-molecule–based superresolution imaging. PNAS..

[102] Los GV, Encell LP, McDougall MG, Hartzell DD, Karassina N, Zimprich C (2008). HaloTag: a novel protein labeling technology for cell imaging and protein analysis. ACS Chem Biol.

[103] Gautier A, Juillerat A, Heinis C, Corrêa IR, Kindermann M, Beaufils F (2008). An engineered protein tag for multiprotein labeling in living cells. Chem Biol.

[104] Shi Y (2014). A glimpse of structural biology through X-ray crystallography. Cell..

[105] Montelione GT, Zheng D, Huang YJ, Gunsalus KC, Szyperski T (2000). Protein NMR spectroscopy in structural genomics. Nat Struct Biol.

[106] Fernandez-Leiro R, Scheres SHW (2016). Unravelling biological macromolecules with cryo-electron microscopy. Nature..

[107] Schuster CF, Wiedemann DM, Kirsebom FCM, Santiago M, Walker S, Gründling A (2020). High-throughput transposon sequencing highlights the cell wall as an important barrier for osmotic stress in methicillin resistant *Staphylococcus aureus* and underlines a tailored response to different osmotic stressors. Mol Microbiol.

[108] Meeske AJ, Rodrigues CDA, Brady J, Lim HC, Bernhardt TG, Rudner DZ (2016). High-throughput genetic screens identify a large and diverse collection of new sporulation genes in *Bacillus subtilis*. PLoS Biol.

[109] Duggan S, Laabei M, Alnahari AA, O’Brien EC, Lacey KA, Bacon L (2020). A small membrane stabilizing protein critical to the pathogenicity of *Staphylococcus aureus*. Infect Immun.

